# Replication of Micro- and Nanofeatures in Injection Molding of Two PLA Grades with Rapid Surface-Temperature Modulation

**DOI:** 10.3390/ma11081442

**Published:** 2018-08-15

**Authors:** Sara Liparoti, Vito Speranza, Roberto Pantani

**Affiliations:** Department of Industrial Engineering, University of Salerno–via Giovanni Paolo II, 132, 84084 Fisciano (SA), Italy; sliparoti@unisa.it (S.L.); rpantani@unisa.it (R.P.)

**Keywords:** replication, microfeature, nanofeature, injection molding, polylactic acid, mold temperature

## Abstract

The production by injection molding of polymeric components having micro- and nanometrical surfaces is a complex task. Generally, the accurate replication of micro- and nanometrical features on the polymeric surface during the injection-molding process is prevented by of the low mold temperature adopted to reduce cooling time. In this work, we adopt a system that allows fast heating of the cavity surface during the time the melt reaches the cavity, and fast cooling after heater deactivation. A nickel insert with micro- and nanofeatures in relief is located on the cavity surface. Replication accuracy is analyzed by Atomic Force Microscopy under different injection-molding conditions. Two grades of polylactic acid with different viscosity have been adopted. The results indicate that the higher the cavity surface temperature is, the higher the replication accuracy is. The viscosity has a significant effect only in the replication of the microfeatures, whereas its effect results are negligible in the replication of nanofeatures, thus suggesting that the interfacial phenomena are more important for replication at a nanometric scale. The evolution of the crystallinity degree on the surface also results in a key factor on the replication of nanofeatures.

## 1. Introduction

Nanoscale technologies, advanced microfabrication, and postprocessing modification techniques support the realization of a wide range of two- and three-dimensional (2D and 3D) objects that can be adopted in several fields, from electronic to biomedical [1,2,3,4,5]. The micro- and nanostructured surfaces can improve cell adhesion during cell growth in tissue engineering [6,7]. Furthermore, they open the possibility to produce surfaces with super-hydrophobic characteristics without additional coating processes [8,9].

The techniques adopted for the production of micro- and nanostructured surfaces can be briefly summarized in two categories: bottom–up and top–down approaches. Bottom–up approaches are related to the construction of micro- and nanostructured materials and devices by the self-assembly of atoms or molecules. The most diffused bottom–up techniques are: the atomic layer deposition [10], sol-gel processes [11], and molecular self-assembling [12]. The top–down approach corresponds to the production of micro- and nanoscaled structures starting from larger dimensions and reducing them to the required values [13]. The most common top–down approaches are lithography-based techniques such as soft lithography, e-beam lithography, and nanoimprint lithography [14,15,16,17]. Both the mentioned approaches require high cost, and long processing times. Furthermore, there is only limited control over surface properties. This makes the application of these techniques difficult in the production of large-area micro- and nanostructured surfaces.

Replication methods can represent an excellent alternative since they couple the high dimensional accuracy of lithography, adopted for the production of the masters to be replicated, with the short processing time of techniques such as embossing and injection molding [18,19,20,21].

The injection-molding process has been adopted for the production of biocompatible and biodegradable materials; thus, this process could also be promising for the production of micro- and nanostructured surfaces made of biomaterials [8,22,23,24,25]. The injection-molding process can be divided in three main stages: The filling, during which the polymer in the molten state fills the cavity having the master to be replicated on its surface; the packing, during which additional material is forced into the cavity to compensate for the shrinkage; and the cooling. The selected mold temperature is generally low, smaller than the glass-transition temperatures for most polymers. The use of low mold temperature reduces the cooling time that represents the most processing time. However, the high cooling rates that the polymer experiences in such conditions induces the formation of a frozen layer at the polymer–mold interface that prevents the accurate replication of the micro- and nanostructures present on the master. To overcome this limitation, many authors have proposed to couple the injection-molding process with a system that allows the rapid control of mold temperature, to carry out the filling and packing with high temperature, and to carry out the cooling with low temperatures. Among these systems, induction heating [26], proximity heating [27], and infrared heating [28] have been proposed in the literature. These techniques suffer from high additional tool costs, and the processing time is significantly longer than the processing time of the conventional injection-molding process. The electrical heating of the cavity surface is undoubtedly less expensive and requires small additional tool costs [29,30,31,32,33]. The electrical heating of the cavity surface was efficiently applied to the injection-molding process to obtain micro- and nanostructures surfaces of polypropylene [34].

In this paper, the injection-molding process, coupled with a system that allows fast evolution of the temperature on the cavity surface, has been adopted to produce micro- and nanostructures objects made of polylactic acid (PLA), a biodegradable and biocompatible polymer. The temperature increase is achieved by the ohmic heating of thin carbon black loaded poly (imide-amide) film. The injection-molding sample has been characterized by morphological analyses and by X-ray. Two different grades of PLA have been adopted to analyze the effect of viscosity on replication ability.

## 2. Materials and Methods

Two grades of commercial PLA, produced by NatureWorks (Minnetonka, MN, USA), have been adopted in this work. PLA with the trade name of 4032D is characterized by about 2% of D-enantiomer, a maximum crystallinity of 45%, a molecular weight M_w_ = 210 kg/mole, and polydispersity index of 1.75. PLA with the trade name of 3251D is characterized by about 1% D-enantiomer content, a molecular weight M_w_ = 90 kg/mole, and a polydispersity index of 1.62. The rheology of both PLA grades is reported elsewhere [35].

A Negri Bossi injection-molding machine (mod. 70ton, Cologno Monzese, Italy) has been adopted to obtain molded samples of two different grades of PLA. The injection molding conditions are: 2.8 cm^3^·s^−1^ average flow rate, 200 °C melt temperature, 8 s holding time, and 63 MPa or 30 MPa as holding pressures. The mold is equipped with 5 pressure transducers located along the flow path, from the nozzle (position P0) to the end of the cavity (P1 in the runner, P2-P3-P4 inside the cavity). A thin gate is adopted upstream a rectangular cavity. A detailed description of the cavity and the positions of pressure transducers along the flow path are reported elsewhere [34].

The cavity surface temperature evolution has been obtained by a heating device layered below the cavity surface. The heating device is composed of a conductive layer of carbon black loaded poly (amide-imide), having 70 µm thickness. The conductive layer is electrically insulated from the mold by Kapton^®^ layers, having thickness of 20 µm. An additional Kapton^®^ insulating layer 140 µm thickness is located between the mold and the heater to reduce heat loss through the mold. A detailed description of the heater is reported elsewhere [36]. A steel layer of 400 µm thickness covers the heating device and protects it from the melt. Heater activation starts at the mold closing time; this time corresponds to 4 s before the melt came in contact with the cavity surface in position P2. The time that the melt reaches position P2 corresponds to t = 0 s for all pressure and temperature evolutions.

A nickel shim, a strip of 400 μm thickness, with micro- and nanocrosses in relief, is located just downstream from the gate in position P2, and replaced a part of the steel layer constituting the cavity surface. Micro- and nanocrosses were produced following a procedure reported elsewhere [37]. Each wing of the microfeatures is 5 µm in height and 20 µm in width. Each wing of the nanofeatures is 60 nm height and 500 nm width.

Table 1 summarizes all the operative conditions. The name of each test contains the constant temperature reached on the cavity surface (T_level_), when the heater is supplied with the correct electrical power, the time that the heater is active, the adopted holding pressure, and the PLA grade (A for PLA3251D and B for PLA4032D).

Atomic Force Microscopy (AFM) allows analyzing the replication accuracy of the samples. The acquisitions have been carried out in the air at room temperature with Dimension 3100 coupled with a Bruker Nanoscope V controller, operating in contact mode. Commercial probe tips have been accurately selected to reduce the discrepancy between the real shape and the acquired one. The selected tip has a spring constant of 1–5 N/m, a radius of 8–12 nm, and height of 10–15 µm. Asymmetric contact angles characterize the tip: the front and the back angles are 25° and 15°, respectively. Five samples for each test were analyzed by AFM and, to reduce distortions, the samples have been rotated, and the acquisitions are referred to a distance from the cross center of 15 µm.

The height AFM patterns have been firstly derived, and then the absolute values of the first derivative have been fitted with the following Equation:

(1) $$y=y_{0}+A\frac{1}{1+e^{-\frac{x-x_{c}+w_{1}/2}{w_{2}}}}\left(1-\frac{1}{1+e^{-\frac{x-x_{c}+w_{1}/2}{w_{3}}}}\right)$$

where *y*_0_ is the offset, *x_c_* is the center, *A* is the amplitude, *w*_1_ is the width at half maximum, and *w*_2_ and *w*_3_ take into account the asymmetry of the curve. The Full Width calculated at the Half Maximum of the fit curve (FWHM) is an index of the channel width that is also adopted as replication accuracy index for the microfeatures [38].

The deviation of the height of the replicated nanofeatures, namely *h_m_*, with respect to the expected value, namely *h_Ni_*, which is the height of the nanofeatures located on the nickel shim, allows quantifying the replication accuracy for the nanofeatures:

(2) $$H=\frac{h_{Ni}-h_{m}}{h_{Ni}}100$$

Injection-molded samples have been analyzed, in the same position where the nickel shim is located, by X-ray Diffractometry (XRD) in reflection mode by an Advance D8 Bruker (Billerica, MA, USA) diffractometer (with a continuous scan attachment and a proportional counter) with Ni-filtered Cu-K_α_ radiation. The full spectrum is considered as a superposition of number of reflections (5 reflections were considered: 2θ = 12.5, 14.7, 16.7, 19.1, and 22.5); each reflection being described by a combination of a Lorentzian function and a Gaussian function [39]. This analysis allows the assessment of the crystallinity degree at the surface of the sample. The same analysis has been repeated after removing 100 µm of polymer at the sample skin. To this purpose, a lapping process is performed by a Mecapol 2B lapping machine (Presi, Eybens, France) using a Mecaprex abrasive disc P1000 (Presi, Eybens, France). The lapping has been performed with a lapping pressure of 50 kPa. The repetition of the X-ray analysis provides thus the value of crystallinity degree at a distance of about 100 µm from the sample surface.

## 3. Results

### 3.1. Temperature and Pressure Evolutions

**Figure 1:**
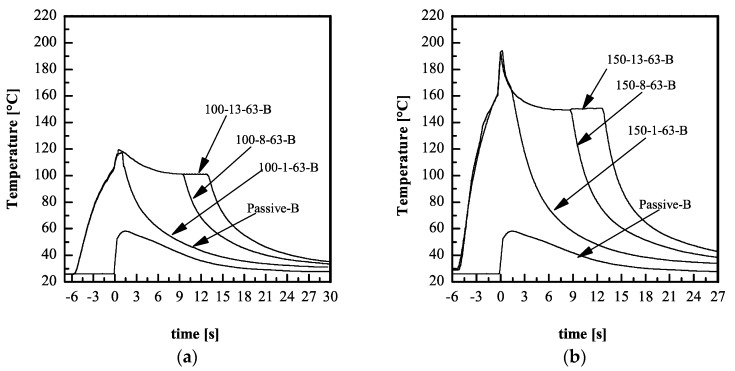
Temperature evolutions recorded during the experiments performed on PLA4032D (**a**) with T_level_ = 100 °C, and (**b**) T_level_ = 150 °C; the temperature evolution of the test Passive-B is also reported for comparison.

Pressure and temperature evolutions give an indication of the thermomechanical history experienced by the polymer during the process. The thermomechanical history influences the replication accuracy. Figure 1 shows the temperature evolutions recorded during the experiments on PLA4032D with different electrical powers, which means different T_level_ (see Table 1).

Temperature on the cavity surface increases from the bulk temperature of the mold, 30 °C, to the temperature T_level_ before the melt reaches the cavity. The contact of the melt with the cavity induces an additional and sharp temperature increase at t = 0 s; after that, the melt starts to cool down to T_level_, and this value is kept constant as long as the heater is active. At heater deactivation, the complete cooling of the polymer takes place. Obviously, when the heater is not activated, the polymer starts to cool down soon after the first contact with the cavity surface. The temperature evolutions recorded during the experiments on the PLA3251D show similar trends.

**Figure 2:**
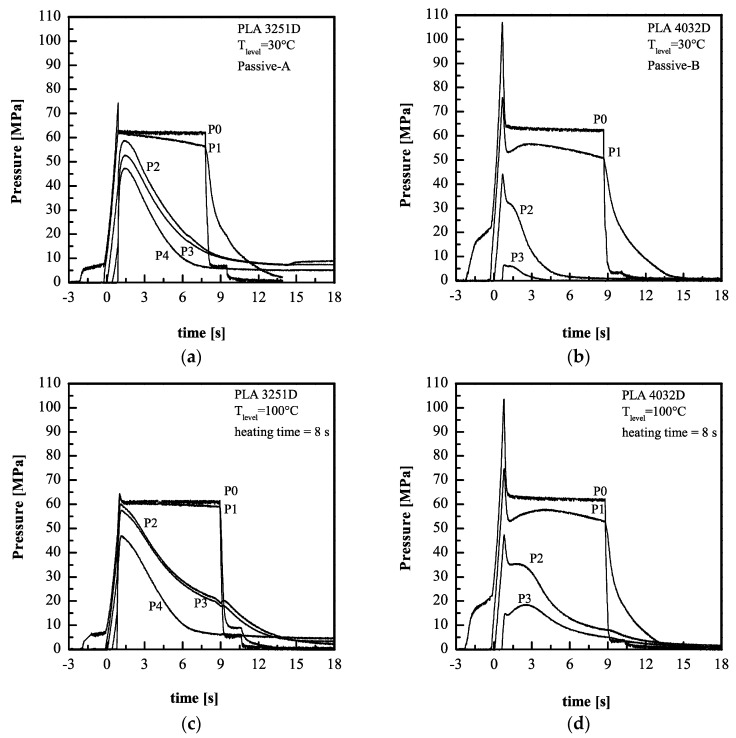

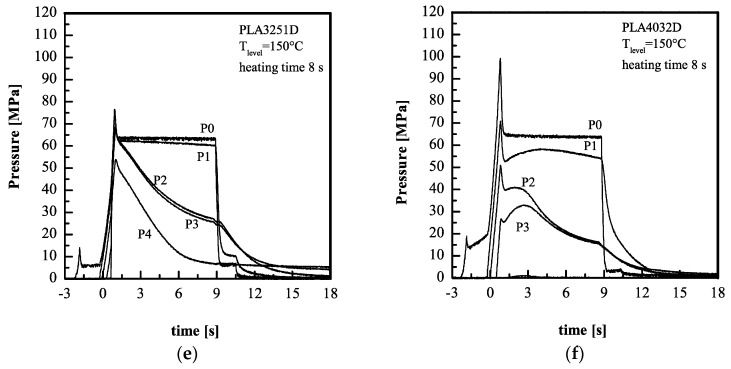
Pressure evolutions in different positions along the flow path recorded during the experiments (**a**) Passive-A, (**b**) Passive-B, (**c**) 100-8-63-A, (**d**) 100-8-63-B, (**e**) 150-8-63-A, and (**f**) 150-8-63-B. In all the figures, t = 0 s corresponds to the time that the melt came in contact with the cavity surface in the position P2.

Figure 2 shows the pressure evolutions along the flow path, recorded during the experiments with two PLA grades and with different T_level_.

The comparison between the temperature evolutions of Figure 1a,b with the pressure evolutions of Figure 2c,f, demonstrates that the temperature on the cavity surface reaches the value T_level_ during the time the melt fills the sprue, and the runner.

The pressure evolutions (see Figure 2) allow identifying the main steps of the injection-molding process: Filling, packing, and cooling. During the filling step, pressure increases in all the positions along the flow path. The pressure peak in position P0 (namely in the nozzle) is due to the filling end and its value decreases with the polymer viscosity; thus, the pressure peak is smaller in the Passive-A case than in the Passive-B case. After the filling, the pressure in position P0 decreases down to the value selected for the packing stage, 63 MPa in the cases shown in the Figure 2. During the packing, additional flow is forced into the cavity to compensate for the shrinkage due to the polymer solidification. This process ends when the gate is sealed. Gate solidification can be detected when the pressure evolution in position P2 presents an inflection point, changing the concavity from downward to upward (Figure 2) [40]. It takes place at about 3 s for all the materials and molding conditions; after this time, the solidification of the polymer takes place along the whole thickness, and pressures in the cavity decrease down to zero for the passive cases (Figure 2a,b). When the heater is active, the cooling takes place in two steps [30] (see Figure 2c–f). The pressure reached after the gate sealing, during the time the heater was active, increased with T_level_. The main difference among the pressure evolutions during the packing for the two PLA grades was related to the pressure drop between P0 and P3, which was significantly higher for PLA4032D than for PLA3251D due to the high viscosity.

A further consideration concerns the pressure evolutions in positions P2 and P3. When PLA3251D was adopted, the pressure in position P3 overlapped the pressure evolutions in position P2; when PLA4032D was adopted, the overlap was not present. The reason for this behavior was due to the polymer viscosity. When PLA3251D was adopted, between positions P2 and P3 the polymer was still in the molten state and, being confined between two sealed parts, the pressure drop between P2 and P3 became negligible [34]. PLA4032D has a higher viscosity and the pressure drop between positions P2 and P3 was still significant; the pressure drop tended to disappear with the increase of T_level_. Additionally, the high viscosity prevented the complete filling of the cavity, and pressure in position P4 was not recorded.

### 3.2. Replication of Microfeatures

**Figure 3:**
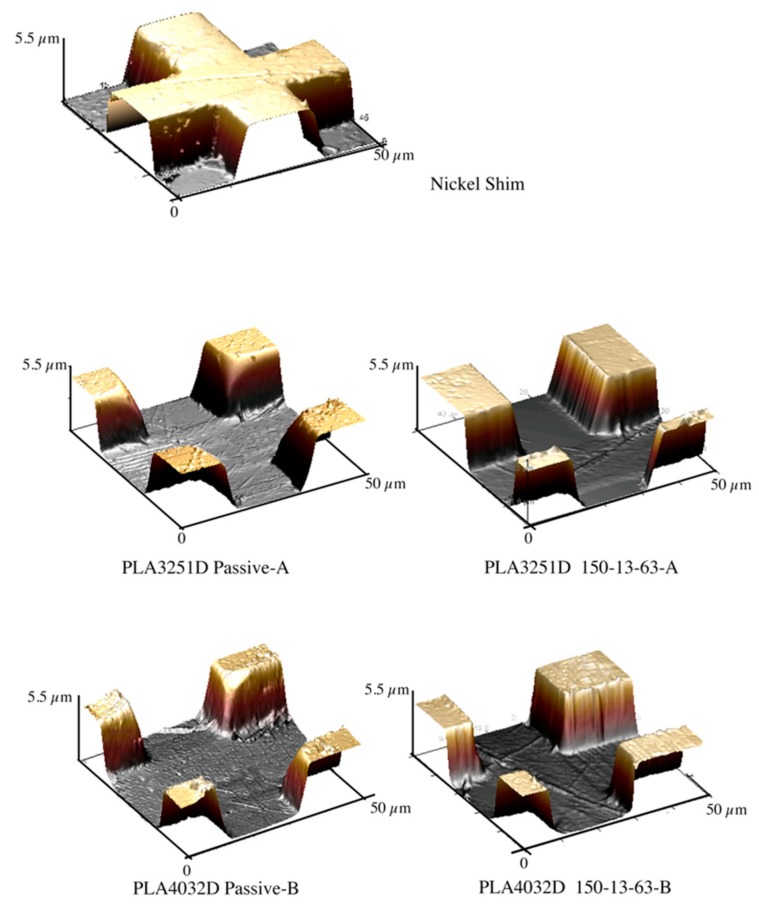
AFM height images of the micro-features on the nickel shim, injection molded samples of PLA3251D and PLA4032D obtained in different conditions of cavity surface temperature.

Figure 3 shows the micro-feature acquired by AFM on the nickel shim and the micro-features acquired on the injection molded samples, with T_level_ = 30 °C (i.e., Passive-A) and with T_level_ = 150 °C kept for 13 s heating time.

**Figure 4:**
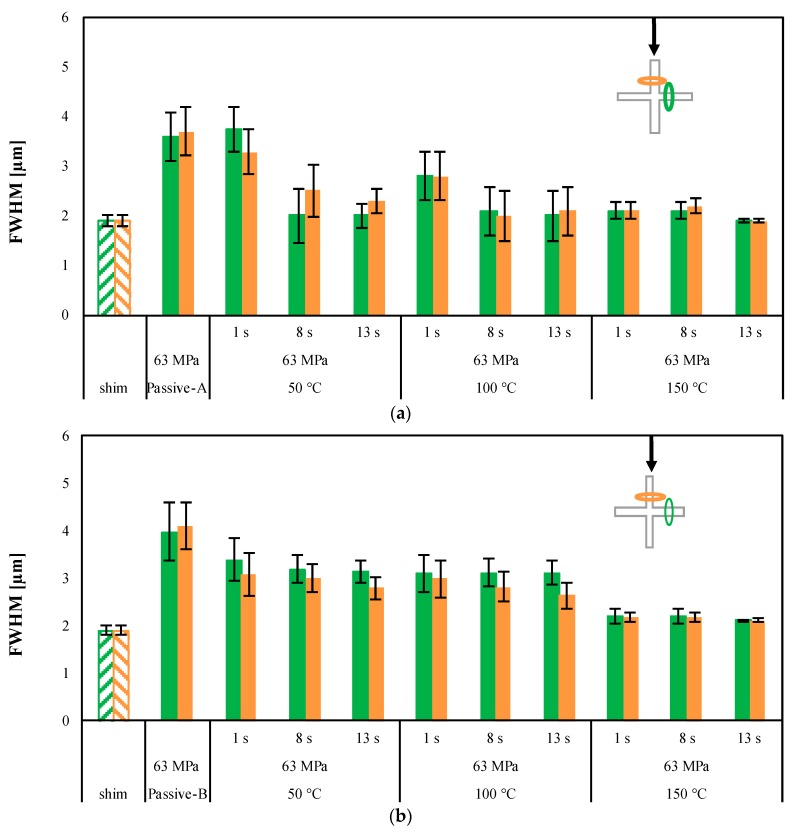
FWHM index of micro-feature on PLA3251D (**a**) and PLA4032D (**b**) samples obtained as indicated in Table 1. A sketch of the area where the AFM patterns have been acquired is also reported.

The samples Passive-A and Passive-B do not show an accurate replication of the micro-feature; indeed, no sharp edge can be detected. The micro-features acquired on the samples 150-13-63-A and 150-13-63-B, obtained with T_level_ = 150 °C, and 13 s heating time, show an accurate replication, as confirmed by the sharp edges. PLA3251D and PLA4032D show a similar dependence of the replicability on the T_level_: Accurate replication is achieved only with high T_level_. For both the PLA grades, the heights of the micro–features are close to the height of the nickel shim with a small deviation, i.e., ±0.05 μm. Figure 4 shows the replication accuracy measured on different injection molded samples in term of FWHM. The values of the FWHM related to the micro-feature on the nickel shim is also reported for comparison.

**Figure 5:**
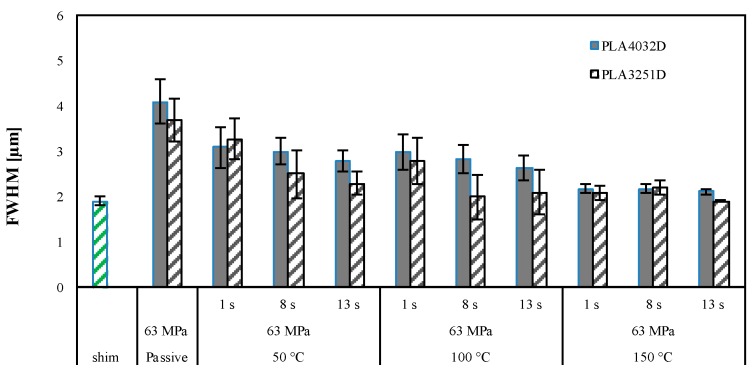
Comparison between the FWHM index, measured in the direction of the flow front, of micro-feature on PLA4032D and FWHM index of micro-features on PLA3251D samples obtained as indicated in Table 1.

For the passive samples, Passive-A and Passive-B, the FWHM is far from the optimum value, 1.97 µm measured on the nickel shim. Figure 4 suggests that the FWHM depends on both the T_level_, and the heating time: the FWHM decreases, namely the replication accuracy increases, as the T_level_ and the heating time increase. When the PLA3251D is adopted, the dependence of FWHM on the cavity surface temperature evolution is significant: with T_level_ = 50 °C and 100 °C, a significant reduction of FWHM can be observed increasing the heating time from 1 s to 8 s. With T_level_ = 150 °C the FWHMs approach values close to that one measured on the nickel shim already with 1 s heating time, and additional decrease with heating time cannot be observed. When PLA4032D is adopted (Figure 4b), the values of FWHM show a smaller dependence on the heating times in all the explored T_level_ ranges. For both the considered materials, the alignment between the wings of the micro-features and the direction of flow does not influence the FWHM values. The micro-features replicated on the PLA4032D show values of FWHM higher than those calculated on PLA3251D. Figure 5 summarizes the differences in replication accuracy, in the direction of the flow front, among PLA3251D and PLA4032D.

**Figure 6:**
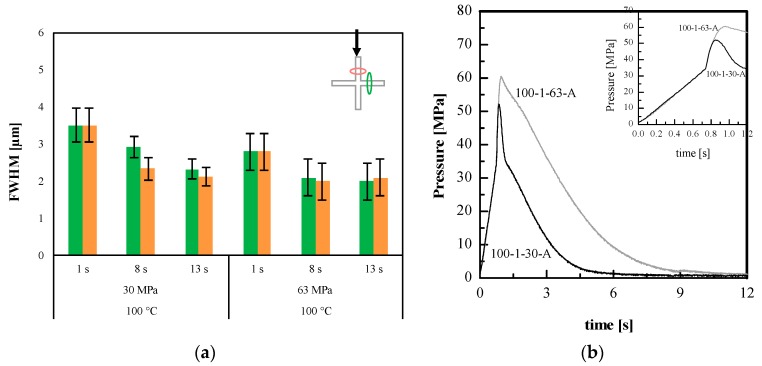
(**a**) FWHM index of micro-feature on PLA3251D samples obtained with different holding pressures and with T_level_ = 100 °C, as indicated in Table 1. A sketch of the area where the AFM patterns have been acquired is also reported. (**b**) Pressure evolutions in position P2 obtained for the experiments 100-1-63-A and 100-1-30-A.

Additional experiments have been carried out also with smaller holding pressure, 30 MPa, to highlight the effect of holding pressure on the replication accuracy. Figure 6a shows the results in term of FWHM for the experiments carried out on PLA3251D sample with T_level_ = 100 °C. Figure 6b shows the pressure evolutions recorded in position P2, where the nickel shim is located, for the experiments 100-1-63-A and 100-1-30-A, carried out with different holding pressure.

Figure 6a shows that the FWHM decreases with the heating time for both the considered holding pressures. Generally, an increase in holding pressure, giving higher pressure levels during the whole process (see Figure 6b), improves replication accuracy. However, Figure 6a shows that the increase of the holding pressure induces only a slightly decrease of the FWHM index. This means that most of the replication process is achieved during the macroscopic cavity filling stage (which does not depend on the holding pressure adopted) that ends at t = 0.8 s. The replication process is completed at the beginning of the packing stage, so that an increase of the packing pressure improves, even if marginally, the replication process.

The replication process can be considered as the filling of micro and nano-cavity by a pressure driven flow [34,41]. During such a flow, the pressure exerted on the polymer has to overcome the pressure drop due to the filling of micro-cavity, the pressure due to the trapped air, and the pressure due to the surface tension [42]. During the filling of micro-cavity, the volume between the feature to be replicated and the polymer undergoes to a reduction. The air that is partially trapped in this volume is compressed. The pressure due to the trapped air, becoming comparable to the pressure exerted on the polymer, delays the filling of the micro-cavity [34]. The pressure due to the surface tension becomes more significant as the dimension of the feature decreases. The pressure due to the surface tension also delays the filling of micro-cavity. Furthermore, during such a pressure driven flow, the polymer undergoes a solidification and a frozen layer is formed at the polymer-air interface. The frozen layer has to elastically deform to allow the replication process to proceed [43]. All these phenomena have to be taken into account in the analysis of the replication accuracy.

**Figure 7:**
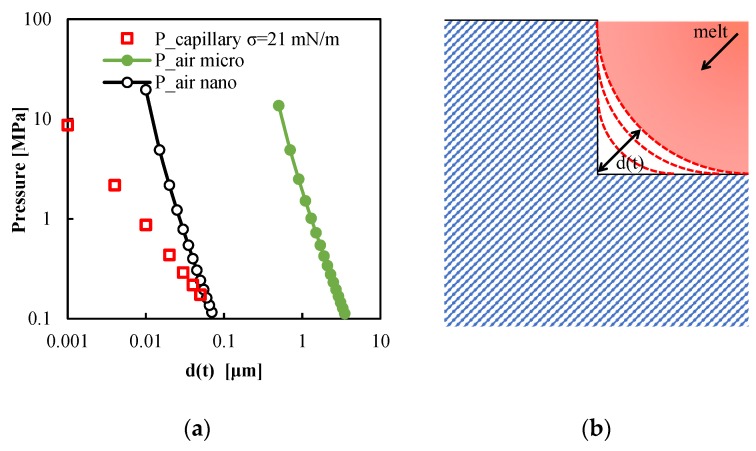
(**a**) Pressure due to the trapped air and to the surface tension as function of the unfilled distance d(t). (**b**) Sketch of the replication process viewed as the filling of a micro-cavity.

To obtain an order of magnitude of the forces involved in the replication process, the melt front has been considered as a cylinder that wets the cavity surface with a contact angle of 180° on both the perpendicular surfaces of the micro or nano-feature (see Figure 7b). The radius of the cylinder (*R*) is initially equal to the height of the feature to be replicated (i.e., 5 µm for the micro-feature), and then the radius decreases with the ongoing of replication process. The distance between the melt front and the corner of the feature is equal to $d=R\left(\sqrt{2}-1\right)$ and the accuracy of the replication increases as *d* decreases. Assuming isothermal conditions, considering air as an ideal gas, the pressure due to trapped air, *P_air_*, is:

(3) $$P_{air}=\frac{V_{0}}{V}P_{0}=\frac{R_{0}^{2}}{R^{2}}P_{0}=\frac{d_{0}^{2}}{d^{2}}P_{0}$$

where *V*_0_ and *P*_0_ are the initial volume and pressure, respectively, and *V* is the current volume.

The pressure due to surface tension is:

(4) $$P_{s}=\frac{\sigma }{R}=\frac{\sigma \left(\sqrt{2}-1\right)}{d}$$

where *σ* is the surface tension (reported in the literature for PLA is 21 mN/m [44]).

Once the two pressures are related to the distance *d*, their value can be calculated as reported in the Figure 7a. Obviously, *P_S_* depends only on *d*, whereas *P_air_* also depends on the initial volume occupied by the gas, in which a pressure *P*_0_ = 1 bar is assumed.

As clear from Figure 7, surface tension should not play any role in the replication of micro-features. When the features have dimensions in the nano-scale, surface tension becomes significant, although the pressure due to trapped air is dominant, if air cannot escape the volume to be replicated.

**Figure 8:**
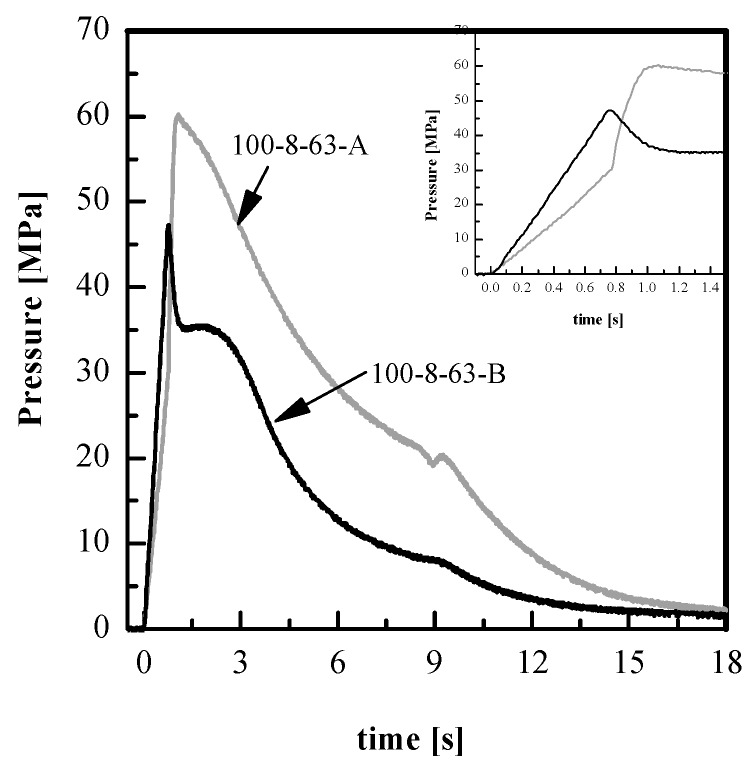
Pressure evolutions in position P2 obtained for the experiments 100-8-63-A and 100-8-63-B.

The pressure exerted on the melt during the filling of the micro-cavity is essentially the pressure recorded in the position *P*2, since in this position, the nickel shim containing the features to be replicated is located. The pressure exerted on the melt depends on the viscosity. The viscosity, on its turn, increases with the molecular weight. Figure 8 shows the influence of the viscosity on the pressure evolution in position *P*2, related to the two PLA grades adopted in this work, with T_level_ = 100 °C. It can be noticed that the pressures reached during the cavity filling (t = 0–0.8 s), reach a value of about 50 MPa for both materials, high enough to overcome the resistance due to *P_air_* and *P_s_* for both micro- and nanofeatures until a reduction of *d* of about one order of magnitude is attained.

The PLA4032D experiences higher pressure during the filling and smaller pressure during the packing than PLA3251D. During the filling (t = 0–0.8 s), the pressure is higher for PLA4032D, than PLA3251D. This is due to the fact that the higher is the viscosity, the higher the pressure required to fill the macroscopic cavity. During the packing stage, the pressure is smaller for PLA4032D, than PLA3251D, since the pressure drop between the injection point and the position inside cavity is higher because the viscosity is higher. Figure 5 shows that the FWHM obtained with 1 s heating time is similar for the two PLA grades, at all the adopted T_level_. This could be due to the fact that the high-pressure levels (certainly higher than those recorded with PLA3251D) recorded with PLA4032D compensates for the pressure required to fill the micro-cavity. With long heating times, the pressure exerted on the polymer during the micro-cavity filling is higher adopting PLA3251D than PLA4032D, thus the replication accuracy is lower for the latter polymer. This also confirms that the replication process completes during the early packing stage.

### 3.3. Replication of Nanofeatures

**Figure 9:**
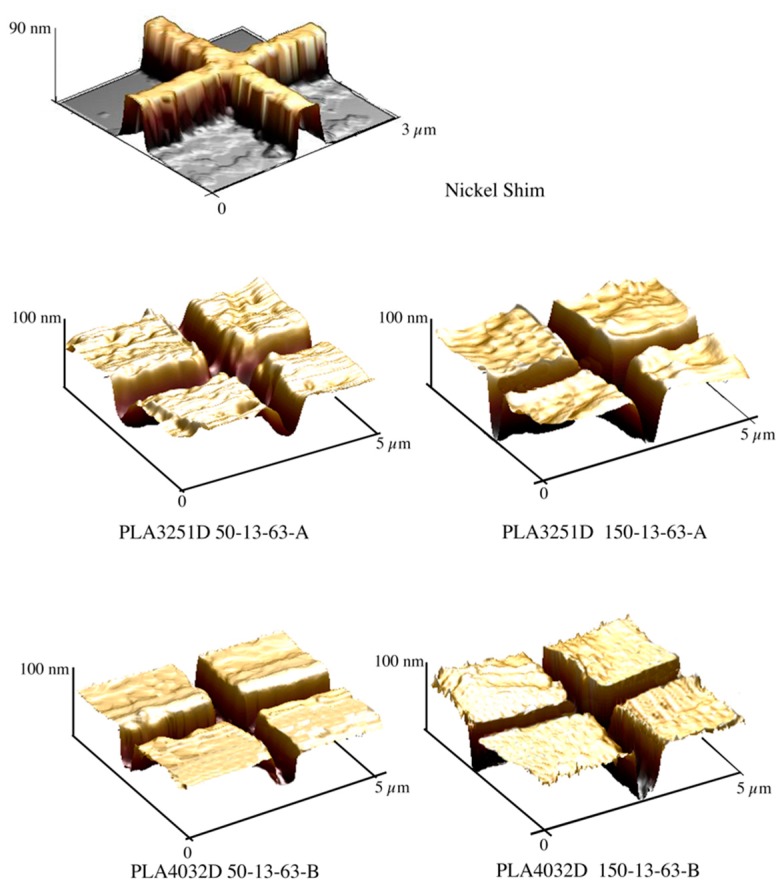
AFM height images of the nanofeatures on the nickel shim, injection-molded samples of PLA3251D and PLA4032D obtained in different conditions of cavity surface temperature.

The replication ability has been also analyzed in the cases of nanofeatures replication. It is important to highlight that nanofeatures have not been found on the passive samples for both PLA grades. Figure 9 shows the AFM acquisitions of the nanofeatures of the nickel shim and the nanofeatures produced during the injection-molding process with two T_level_, 50 °C and 150 °C, and 13 s heating time.

**Figure 10:**
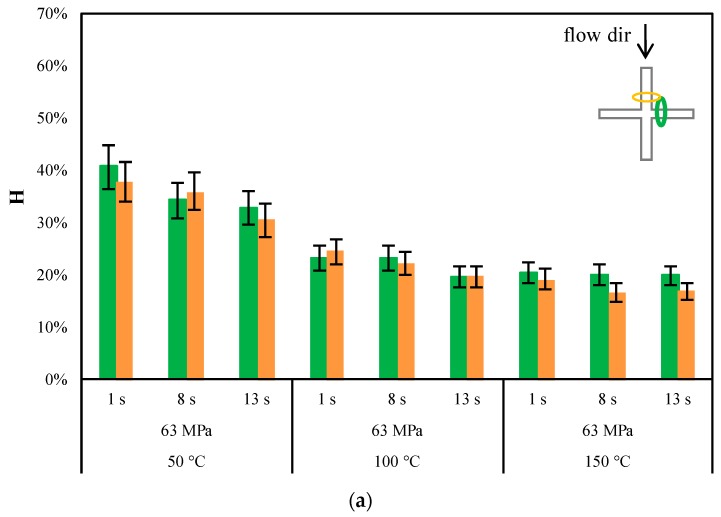

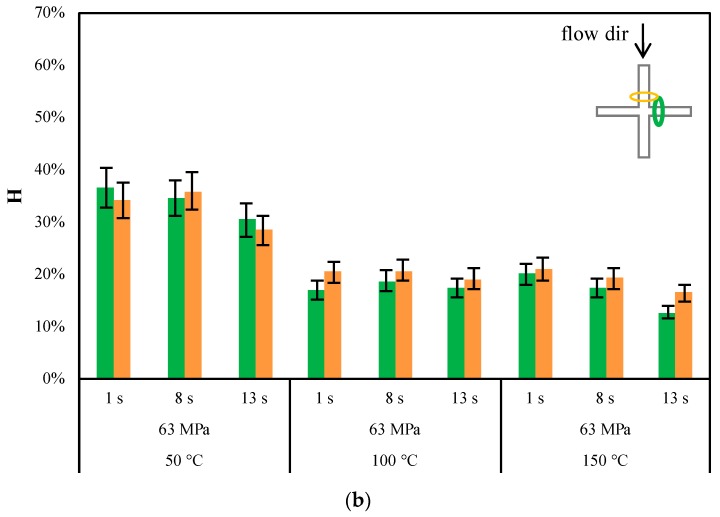
H index of nanofeatures on (**a**) PLA3251D samples and (**b**) PLA4032D samples obtained as indicated in Table 1. A sketch of the area where the AFM patterns have been acquired is also reported.

**Figure 11:**
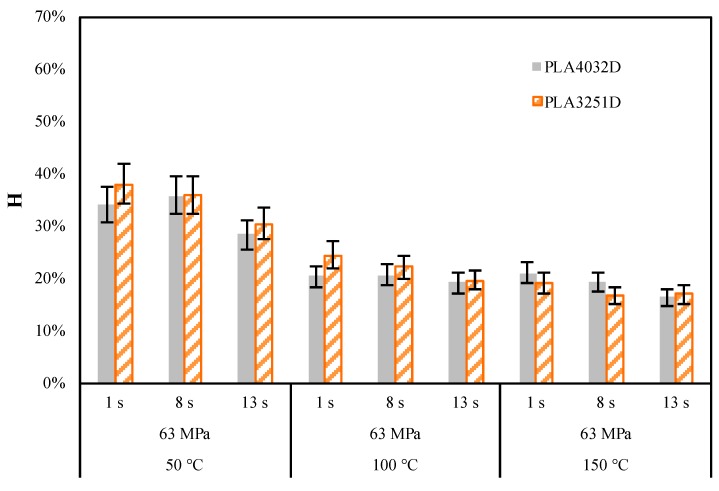
Comparison between the H index of nanofeatures on PLA3251D and H index of nanofeatures on PLA4032D samples obtained as indicated in Table 1.

Figure 10a,b shows that the replication accuracy does not depend on the direction of nanofeatures (any difference is within the confidence range of the measurement) with respect the direction of the flow front for both the PLA grades. Furthermore, the higher T_level_ is, the smaller the H index is, for both PLA grades. Figure 11 summarizes the differences in replication accuracy in terms of H index, in the direction of the flow front, between PLA3251D and PLA4032D.

The AFM acquisitions reported in Figure 9 show that replication accuracy was poor with T_level_ = 50 °C. The height of the nanofeature replicated on the polymer is significantly smaller than that on the nickel shim. The higher is the T_level_, the closer is the height of the replicated nanofeature to the height measured for the nanofeature on the nickel shim. A quantitative analysis of the replication accuracy is given in terms of H index in the Figure 10, for both the adopted PLA grades.

First of all, Figure 11 shown that good replication has been reached for both PLA grades: the value of the H index is 16 ± 2% (i.e., the height of the replicated nanofeature is 50 ± 1 nm) with T_level_ = 100 °C.

The heating time seems to have had only a slight effect on nanofeature-replication accuracy. Furthermore, replication accuracy seems to be not dependent on the PLA grade. These observations suggest that the replication mechanism is less dependent on viscosity with respect to the cases of the microfeatures. The phenomena relating to the interaction between the polymer and the cavity surface, the nickel shim in this work, become more important as the dimension of the feature to be replicated decreases down to a nanometrical level.

### 3.4. Analysis of Morphology

**Figure 12:**
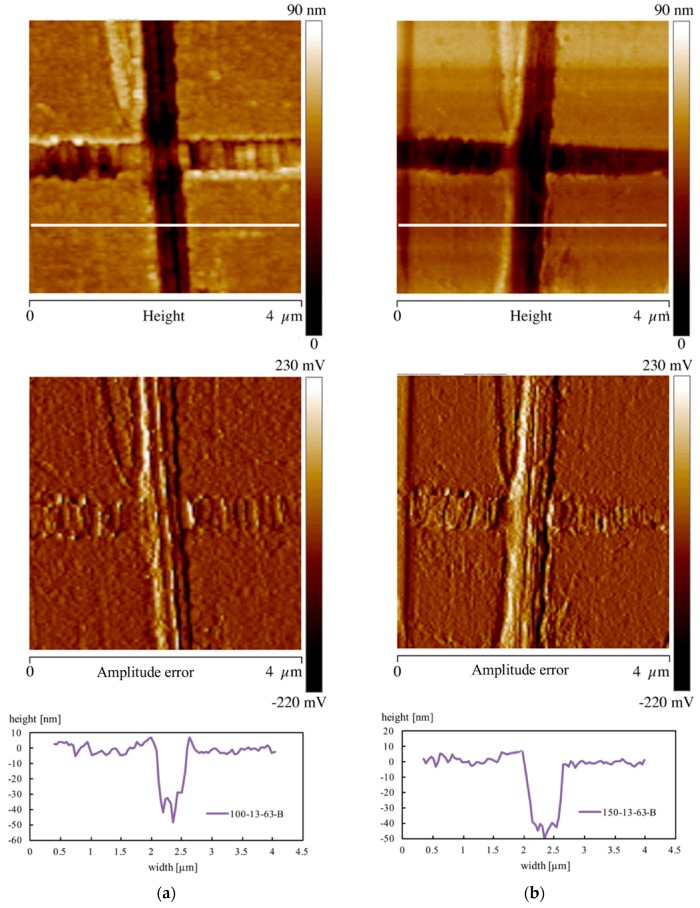
Height and amplitude error AFM maps of the samples (**a**) 100-13-63-B and (**b**) 150-13-63-B. The pattern related to the white line reported in the height map is also shown.

Morphological analysis has been also performed on the molding surface in the areas where nanofeatures have been found. Figure 12 shows the height and amplitude error AFM acquisitions related to the samples 100-13-63-B and 150-13-63-B. The cross sections of the height maps were also reported for each sample.

The AFM maps show that the samples produced activating the heating device are characterized by the presence of structures aligned with the flow direction. The pattern of the cross sections reported in the bottom of the figure shows that the height of the structures, 10 ± 5 nm, is comparable with the height of the features to be replicated. Similar patterns have been also obtained adopting PLA3251D with the same T_level_ and heating time. Thus, the H value obtained at 100 °C and 150 °C T_level_ cannot further decrease at values smaller than 16% because of the presence of the structures aligned with the flow front.

**Figure 13:**
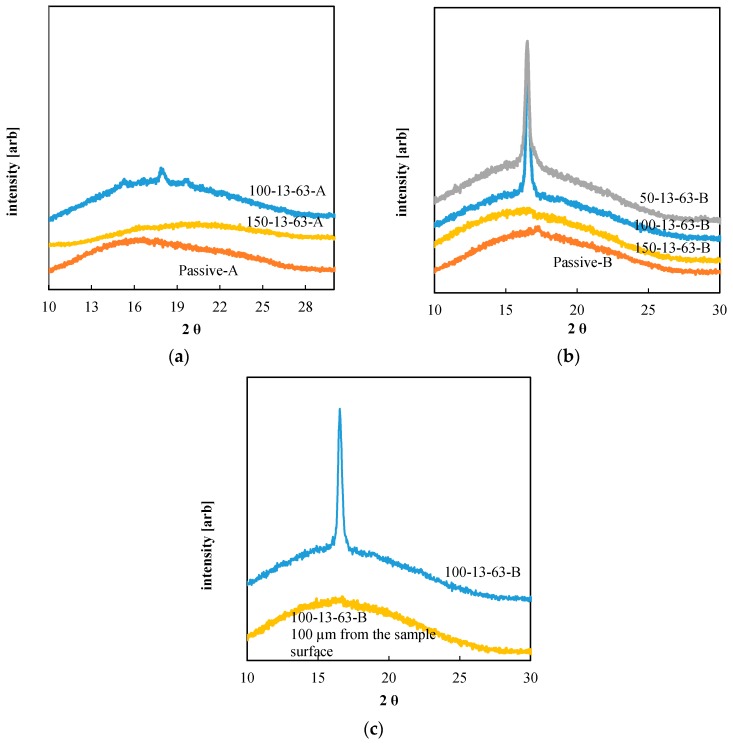
X-ray Diffractometry (XRD) spectra of the PLA injection-molding samples (**a**) PLA3251D, and (**b**) PLA4032D. (**c**) XRD spectra along the sample thickness for the sample 100-13-63-B.

The XRD analysis performed on the surface of the injection-molded samples is reported in the Figure 13.

The samples obtained with low cavity-surface temperature, Passive-A and Passive-B, show a low crystalline degree due to the high cooling rates that the polymer experiences during the process; the samples obtained with T_level_ = 150 °C also show a low crystallinity degree. The samples obtained with T_level_ = 100 °C show the highest crystallinity degree (see the peak at 2θ ≅ 16.7°), 5 ± 3% for PLA3251D, and 10 ± 3% for PLA4032D. Such crystalline degrees are significant for PLA, since the maximum value of the crystallinity degree of these thermoplastic materials is about 40% [45]. Figure 13b shows that there was a shift of the peak 2θ ≅ 16.7° (attributed to the α-phase [46,47]) toward high values of 2θ with the decrease of T_level_. This shift is due to the different cooling and flow conditions that the polymer experiences during the solidification that took place with different T_level_ and heating times [47,48,49].

The crystalline degree found for both the PLA grades was also consistent with the presence of structures aligned with the flow front and shown the Figure 12. Figure 13c shows the XRD spectra of the sample 100-13-63-B on the sample surface and 100 µm distance from the sample surface. The comparison between the two spectra demonstrates that the high crystallinity was limited to a thin layer close to the sample surface. This suggests that the formation of the structures aligned with the flow front was also limited to a narrow layer close to the sample surface.

It is worth mentioning that the PLA grades adopted in this work, and generally all the commercial grades of PLA, had crystallization times (in quiescent conditions) much longer than the processing times of injection molding. This means that the crystalline structures detected in our samples had to be due to flow-induced crystallization, and their fibrillary morphology confirms this interpretation [50].

## 4. Discussion

In this work, the replication of micro- and nanofeatures on two different grades of PLA during the injection-molding process has been experimentally analyzed. The system adopted to modulate the temperature on the cavity surface during the injection-molding process has been demonstrated as efficient in the enhancing of replication accuracy of both micro- and nanofeatures.

The replication accuracy of microfeatures increases with both T_level_ and heating time. An increase of packing pressure only marginally improves the replication. The replication is more accurate when the polymer with low viscosity is adopted. The results suggest that the replication process has to take place during both the filling (referred to the macrocavity), and the early packing. When the heating time is comparable with the filling time of the macroscopic cavity, the high-pressure levels recorded with PLA4032D, the high viscosity polymer compensates for the pressure required to fill the microcavity. The frozen layer, which starts to form immediately after heater deactivation, prevents any additional filling of the microcavity during the packing stage. As a result, the differences observed in the replication accuracy with 1 s heating time are within the confidence range of the measurement (see Figure 5). Adopting heating times longer than the filling time, significant pressure is exerted on the polymer for longer, and the formation of the frozen layer, because the high T_level_ adopted, is significantly delayed. As a consequence, an increase in replication accuracy with heating time is observed. Additionally, thanks to the smaller viscosity, which reduces the pressure drop at the flow front, PLA3251D shows higher replication accuracy than PLA4032D when heating times longer than filling times are adopted. The interpretation of the replication process described above implies that for the microcavities the replication process can be considered as a viscous filling, for which the interfacial phenomena can be neglected.

In the cases of nanofeatures, the replication accuracy is poorly dependent on the heating time and on the polymer grade. This suggests that the replication mechanism has to be different from the one hypothesized for the replication of microfeatures. In particular, interfacial phenomena become more significant the smaller the dimension of the feature to be replicated is. Interestingly, the AFM maps show the presence of structures aligned along the flow direction. The presence of these structures is consistent with the crystallization degree found on the sample surface. The mean dimension of the structures is in the range of few nanometers and their presence certainly prevents a further increase of the replication accuracy.

## Figures and Tables

**Table 1:** Operating conditions adopted for the injection molding experiments with the heating device used to tune the temperature on the cavity surface (T_level_ is the temperature reached on the cavity surface thanks to the activations of the heating device).

Test Name	Polylactic Acid (PLA) Grade	Holding Pressure [MPa]	Electrical Power [W/cm^2^]	T_level_ [°C]	Heating Time [s]
Passive-A	3251D	63	0	30	0
Passive-B	4032D	63	0	30	0
100-1-30-A	3251D	30	5	100	1
100-8-30-A	3251D	30	5	100	8
100-13-30-A	3251D	30	5	100	13
50-1-63-A	3251D	63	2	50	1
50-8-63-A	3251D	63	2	50	8
50-13-63-A	3251D	63	2	50	13
100-1-63-A	3251D	63	5	100	1
100-8-63-A	3251D	63	5	100	8
100-13-63-A	3251D	63	5	100	13
150-1-63-A	3251D	63	10	150	1
150-8-63-A	3251D	63	10	150	8
150-13-63-A	3251D	63	10	150	13
50-1-63-B	4032D	63	2	50	1
50-8-63-B	4032D	63	2	50	8
50-13-63-B	4032D	63	2	50	13
100-1-63-B	4032D	63	5	100	1
100-8-63-B	4032D	63	5	100	8
100-13-63-B	4032D	63	5	100	13
150-1-63-B	4032D	63	10	150	1
150-8-63-B	4032D	63	10	150	8
150-13-63-B	4032D	63	10	150	13

## Supplementary Materials

Supplementary File 1
