# Supplementary material for: Replication of Micro- and Nanofeatures in Injection Molding of Two PLA Grades with Rapid Surface-Temperature Modulation
Source: Materials (Basel). 2018 Aug 15;11(8):1442. doi: 10.3390/ma11081442 (PMC6119903; doi:10.3390/ma11081442)
Supplement: Supplementary File 1 [file materials-11-01442-s001.pdf]

Materials **2018**, *11*(8), 1442; doi:10.3390/ma11081442

Article

Replication of Micro- and Nanofeatures in Injection Molding of Two PLA Grades with Rapid Surface-Temperature Modulation

Sara Liparoti, Vito Speranza * and Roberto Pantani

1. Supporting material

In the Figure 1S, we report some pictures in polarized light of full-thickness parts of the samples in the region where the features to be replicated are located. It can be noticed that the passive samples show the typical fringes due to high orientation. On increasing the surface temperature, the fringes disappear and the samples become homogeneous. It is interesting to notice that for 3251D a surface temperature of 50 °C is already able to clear all the fringes, whereas this happens for 4032D only with a surface temperature of 100 °C. This is clearly due to the higher relaxation time of this latter grade of PLA.

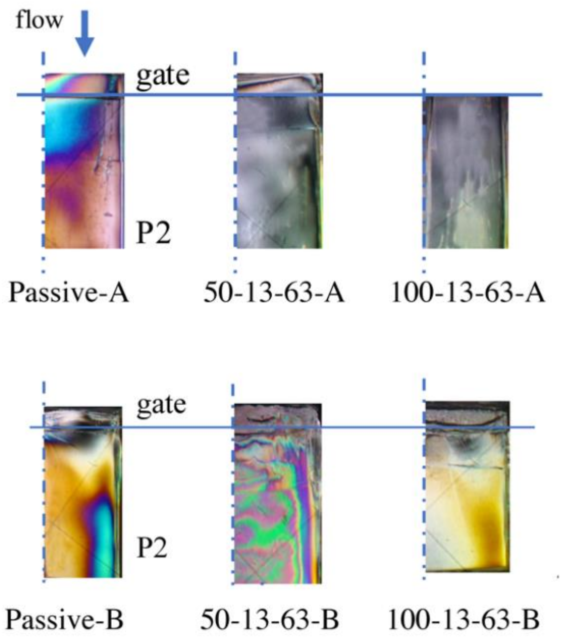

Figure 1S: Pictures in polarized light of full-thickness parts of the samples in the region where the features to be replicated are located.
